# The Authors’ Reply

**DOI:** 10.30476/dentjods.2024.50388

**Published:** 2025-03-01

**Authors:** Mohamad Reza Golzar Feshalami, Mehraban Shahi, Nasrin Davari Dolatabadi

**Affiliations:** 1 Student Research Committee, Faculty of Medicine, Hormozgan University of Medical Sciences, Bandar Abbas, Iran; 2 Health Information Management, Dept. of Health Information Technology, School of Allied Medical Sciences, Hormozgan University of Medical Sciences, Bandar Abbas, Iran

## Dear Editor

We appreciate the insightful feedback on our article discussing the complexities and potential innovations in diagnosing odontogenic tumors through artificial intelligence (AI). The commenter rightly points out the need for a more detailed exploration of specific types of odontogenic tumors that present significant diagnostic challenges. While our article aimed to provide a broad overview of the diagnostic complexities across various tumor types, we acknowledge that a deeper dive into individual tumor characteristics and diagnostic hurdles would have enriched the discussion.

Moving forward, we recognize the importance of focusing research efforts on developing AI algorithms that can effectively differentiate between different types of odontogenic tumors based on their unique clinical and radiographic features. By integrating advanced imaging modalities and machine learning algorithms, researchers can indeed enhance diagnostic accuracy and efficiency, thereby improving patient outcomes.

Additionally, we agree with the commenter’s recommendation regarding the necessity for healthcare professionals to receive adequate training in AI technology. Collaboration between dental practitioners and AI specialists is essential for harnessing the full potential of AI in improving diagnostic precision and streamlining treatment planning for odontogenic tumors.

Moreover, we appreciate the suggestion to explore interdisciplinary approaches by integrating AI with genomics and proteomics. This holistic approach holds promise for advancing our understanding of the molecular mechanisms underlying odontogenic malignancies, which could lead to more targeted and effective therapeutic strategies in the future.

In conclusion, we thank the commenter for their constructive critique and valuable recommendations. Moving forward, we are committed to addressing these points in future research endeavors to further advance AI-driven innovations in the diagnosis and management of odontogenic tumors.

